# A Systematic Approach to Assess the Activity and Classification of PCSK9 Variants

**DOI:** 10.3390/ijms222413602

**Published:** 2021-12-18

**Authors:** Kepa B. Uribe, Kevin Chemello, Asier Larrea-Sebal, Asier Benito-Vicente, Unai Galicia-Garcia, Steeve Bourane, Ali K. Jaafar, Gilles Lambert, César Martín

**Affiliations:** 1 Department of Molecular Biophysics, Biofisika Institute, University of Basque Country and Consejo Superior de Investigaciones Científicas (UPV/EHU, CSIC), 48940 Leioa, Spain; kbelloso@cicbiomagune.es (K.B.U.); alarrea040@ikasle.ehu.eus (A.L.-S.); asierbenitovicente@gmail.com (A.B.-V.); u.galiciag@gamail.com (U.G.-G.); 2 Center for Cooperative Research in Biomaterials (CIC biomaGUNE), Basque Research and Technology Alliance (BRTA), 20014 Donostia San Sebastian, Spain; 3 Inserm, UMR 1188 Diabète Athérothrombose Thérapies Réunion Océan Indien (DéTROI), Université de La Réunion, 97400 Saint-Denis de La Reunion, France; kchemello@gmail.com (K.C.); steeve.bourane@inserm.fr (S.B.); ali.jaafar@univ-reunion.fr (A.K.J.); 4 Fundación Biofisika Bizkaia, 48940 Leioa, Spain; 5 Department of Biochemistry and Molecular Biology, Universidad del País Vasco UPV/EHU, 48080 Bilbao, Spain

**Keywords:** PCSK9, LDL, cholesterol, dyslipidaemias, lipoproteins, receptors, gain of function, loss of function, in vitro characterization, familial hypercholesterolemia

## Abstract

Background: Gain of function (GOF) mutations of PCSK9 cause autosomal dominant familial hypercholesterolemia as they reduce the abundance of LDL receptor (LDLR) more efficiently than wild-type PCSK9. In contrast, PCSK9 loss of function (LOF) variants are associated with a hypocholesterolemic phenotype. Dozens of PCSK9 variants have been reported, but most remain of unknown significance since their characterization has not been conducted. Objective: Our aim was to make the most comprehensive assessment of PCSK9 variants and to determine the simplest approach for the classification of these variants. Methods: The expression, maturation, secretion, and activity of nine well-established PCSK9 variants were assessed in transiently transfected HEK293 cells by Western blot and flow cytometry. Their extracellular activities were determined in HepG2 cells incubated with the purified recombinant PCSK9 variants. Their binding affinities toward the LDLR were determined by solid-phase immunoassay. Results: LDLR expression increased when cells were transfected with LOF variants and reduced when cells were transfected with GOF variants compared with wild-type PCSK9. Extracellular activities measurements yielded exactly similar results. GOF and LOF variants had increased, respectively reduced, affinities for the LDLR compared with wild-type PCSK9 with the exception of one GOF variant (R218S) that showed complete resistance to inactivation by furin. All variants were expressed at similar levels and underwent normal maturation and secretion patterns except for two LOF and two GOF mutants. Conclusions: We propose that transient transfections of HEK293 cells with a plasmid encoding a PCSK9 variant followed by LDLR expression assessment by flow cytometry is sufficient to reliably determine its GOF or LOF status. More refined experiments should only be used to determine the underlying mechanism(s) at hand.

## 1. Introduction

Since the discovery of mutations in the proprotein convertase subtilisin/kexin type 9 (*PCSK9*) gene in French families [1] and its identification as the third genetic cause of autosomal dominant familial hypercholesterolemia (FH) [2], PCSK9 has become an attractive therapeutic target for the prevention of hypercholesterolemia and cardiovascular disease (CVD) [3,4].

PCSK9 is highly expressed by the liver and to a much lesser extent by the intestine [5]. The 22-kb *PCSK9* gene encodes a 692 amino acid protein [6], which is initially expressed as a precursor proPCSK9 (75 kDa) that becomes mature following intramolecular autocatalytic cleavage. Once cleaved, the prodomain remains non-covalently attached to the catalytic domain of PCSK9, allowing the secretion of the mature protein as a catalytically inactive PCSK9-prodomain complex [7]. PCSK9 binds to the epidermal growth factor precursor homology domain A (EGF-A) of the LDLR extracellularly, and the LDLR/PCSK9 complex enters the endosomal pathway [8]. The affinity between PCSK9 and the receptor is increased at the acidic pH of endosomes, which locks the LDLR in an open conformation that drives the PCSK9–LDLR complex to the lysosomal compartment for degradation [8,9,10]. PCSK9 can also interact with the LDLR intracellularly in ER or post-ER compartments and promote intracellular trafficking of the receptor through the trans-Golgi network (TGN) to lysosomes [11].

Large-scale cohort studies have shown the existence of common genetic variants with increased PCSK9 function (gain of function, GOF) as well as loss of function (LOF) PCSK9 variants [12,13]. PCSK9 GOF variants reduce LDL removal from the circulation and therefore are associated with increased circulating LDL cholesterol (LDL-C) levels [14]. Conversely, PCSK9-LOF variants enhance LDL plasma clearance and thereby lower levels of LDL-C and reduce CVD risk [12]. PCSK9 GOF and LOF variants are broadly distributed along the prodomain, catalytic domain and, C-terminal domains of the protein. The mechanisms underlying the effects of these variants are highly heterogeneous. These two facts make it difficult to explain why missense mutations on PCSK9 can either increase or decrease plasma LDL-C levels.

Despite the existence of modern bioinformatics tools [15], in silico predictions often fail to accurately determine the pathogenicity of PCSK9 variants [16]. Only a minority of PCSK9 variants reported to date have been thoroughly studied and genuinely proven to be GOF or LOF variants. In addition to this low frequency of studied variants, the different techniques that have been developed by us and others in the past are extremely heterogeneous [17,18,19,20,21,22] and did not allow head-to-head comparisons of all of these variants.

The purpose of this study was to set up a comprehensive step-by-step systematic methodology to characterize the activity and kinetic parameters of PCSK9 variants in vitro, allowing their determination as GOF or LOF as well as their mechanisms of action. A series of well-characterized PCSK9 variants were selected to validate this approach, and their mechanisms of action were further characterized by determining their affinity for the LDLR and their ability to act on the receptor intracellularly. In this study, we combined in vitro assays using cells transiently expressing PCSK9 variants followed by flow cytometry analyses and cells treated with purified recombinant PCSK9 variants as well as a solid-phase binding immunoassays for the accurate assessment of GOF and LOF status.

## 2. Materials and Methods

### 2.1. Site-Directed Mutagenesis and Cloning

**Figure 1:**
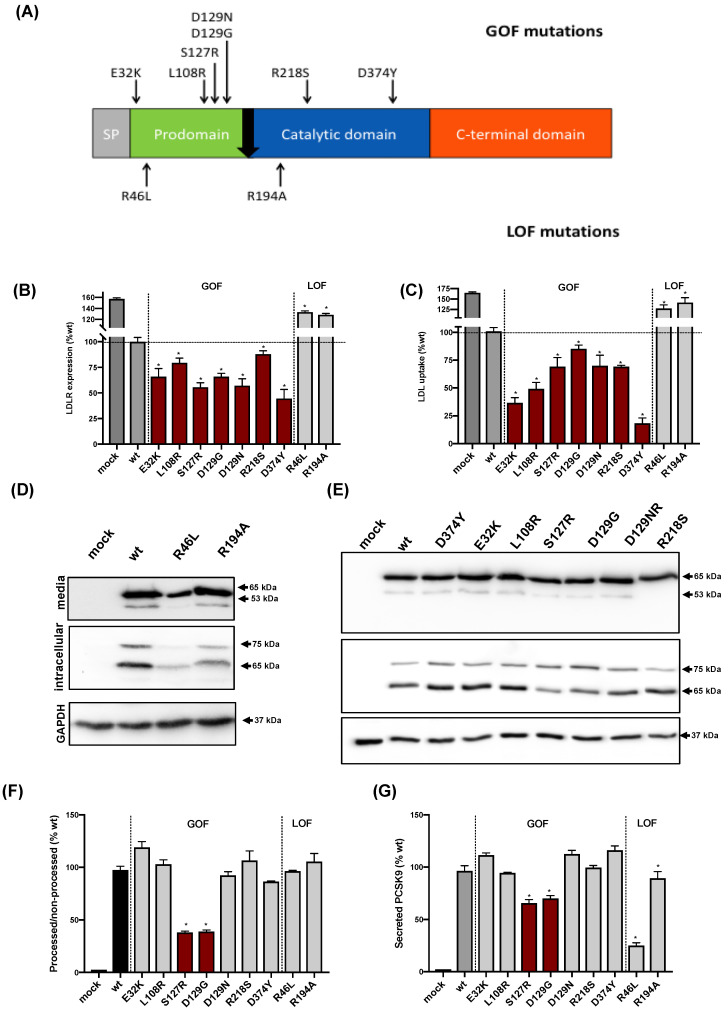
Cell surface LDLR expression, LDL cellular uptake, intracellular expression, secretion, and function of wild-type (WT), LOF, and GOF PCSK9 variants in stably transfected HEK293 cells. (**A**) Relevant structural and functional domains of PCSK9 and location of the mutations included in this study. (**B**) Cell surface LDLR expression determined by flow cytometry. (**C**) LDL cellular uptake measured by flow cytometry. (**D**,**E**) Representative immunoblots of the expression and secretion into the media of WT, LOF, and GOF PCSK9 variants determined by Western blot. (**F**) Ratio between processed (mature) and non-processed (immature) PCSK9 of each variant quantified by densitometry analysis. (**G**) Amount of secreted PCSK9 determined as the ratio between media/intracellular (mature) signals quantified by densitometry. Histograms represent the mean ± SD of three independent experiments. * *p* < 0.01 compared to wild-type PCSK9.

To establish a comprehensive evaluation of PCSK9 variants, we used a series of nine known GOF and LOF variants (Figure 1A). HEK293 cells were stably transfected with the following PCSK9 variants (GOF: E32K, L108R, S127R, D129G, D129N, R218S, and D374Y; LOF: R46L, R194A). Of note, the R194A variant has been generated to characterize the binding motifs of PCSK9 to the LDLR [23] and never described as naturally occurring.

*PCSK9* variants were constructed by Innoprot (Derio, Spain) introducing the variations by oligonucleotide site-directed mutagenesis (QuickChange Lightning mutagenesis kit; Agilent, Santa Clara, CA, USA) into the human *PCSK9* cDNA (NM_174936.3) in a mammalian *wt-PCSK9* expression vector (pCMV-PCSK9-FLAG) kindly provided by Prof. Horton [24]. A FLAG epitope (DYKDDDDK) and a 6x His tag were introduced after the PCSK9 C-terminal domain to allow purification by Immobilized Metal Affinity Chromatography. Direct sequence analysis was used to verify the sequence of each construct.

### 2.2. Cell Cultures and Transient Transfections

HEK293 cell line was grown in DMEM (glucose 1 g/L, Merck, Sigma-Aldrich, Darmstadt, Germany) supplemented with 10% (*v/v*) inactivated fetal bovine serum (FBS) (Thermo Fisher Scientific, Carlsbad, CA, USA), 100 U/mL penicillin, 100 μg/mL streptomycin, and 4 mM glutamine (Thermo Fisher Scientific, Invitrogen, Carlsbad, CA, USA). HEK293 cells, 5 × 10^5^ cell/well in 6-well culture plates (Sarstedt, Hildesheim, Germany), were transfected with 2 µg cDNA of the plasmid carrying the different *PCSK9* variants using the calcium phosphate transfection method. Similar transfection efficiency was confirmed by transfecting in parallel a plasmid encoding a green fluorescent protein.

### 2.3. qRT-PCR and ELISA

HEK293 transfected with the PCSK9 variants were harvested and mRNA was isolated with TRIzol™ Reagent (Invitrogen, Thermo Fisher Scientific, Carlsbad, CA, USA). cDNA was synthesized from 40 ng of RNA using a One-Step SYBR^®^ Primescript™ RT-PCR kit (Takara Bio Inc., Shiga, Japan) on a BioRad C1000™ Cycling Platform. *PCSK9* mRNA levels were normalized to *GAPDH*. Primers used for PCSK9 were: forward 5′-AGGGGAGGACATCATT GGTG-3′ and reverse 5′-CAGGTTGGGGGTCAGTACC-3′, those for GAPDH were: forward 5′-GGAGCGAGATCCCTCCAAAAT-3′ and reverse 5′-GGCTGTTGTCATACTTCTCATGG-3′. Total PCSK9 concentrations in cell lysates and culture media were determined using the Human Proprotein Convertase 9 ELISA Kit (DEIA2677) (CD Creative Diagnostics, London, UK).

### 2.4. Western Blots

First, 48 h after transfection, supernatants of HEK293 cultured cells were collected and cells were lysed to determine secreted and intracellular PCSK9 levels by Western blot. Proteins from cell lysates or the supernatants were resolved by 8.5% Tris-Glycine SDS-PAGE, and gels were blotted onto nitrocellulose membranes (Protran BA 83, Whatman™, GE Healthcare, Munich, Germany), blocked for 1 h in TBS-T (50 mM Tris-HCl, pH 7.5, 150 mM NaCl, 0.1% Tween 20) containing 5% BSA, and immunoblotted with a mouse-anti-FLAG antibody (DYKDDDK tag rat monoclonal antibody, L5 clone; 1:1000 dilution) (Cat. No: MAI-142; Thermo Fisher Scientific, Invitrogen, Carlsbad, CA, USA) for 16 h at 4 °C. Detection was performed using a horseradish peroxidase-conjugated anti-rat antibody (Cat.No: 7077; Cell Signaling Technology^®^ Inc., Danvers, MA, USA). Proteins were visualized using SuperSignal West Dura Extended Substrate (Thermo Fisher Scientific, Pierce, Carlsbad, CA, USA) on a ChemiDoc XRS apparatus (Bio-Rad, Hercules, CA, USA). Protein quantification was determined relative to glyceraldehyde 3-phosphate dehydrogenase (GAPDH, 1:1000 dilution) (Cat. No.: sc-26778, Santa Cruz Biotech. Inc., Dallas, TX, USA) using the NIH ImageJ software (https://rsbweb.nih.gob/ij/ (accessed on 12 January 2021)).

### 2.5. Recombinant PCSK9 Variants

Sub-confluent HEK293 cells transfected with the plasmids encoding the PCSK9 variants were selected with geneticin (G418 sulfate 0.5 mg/mL) (Thermo Fisher Scientific, Gibco, Carlsbad, CA, USA). PCSK9 purification has been described elsewhere in detail [21]. PCSK9 variants were stored at −80 °C in 50 mM Tris-HCl buffer supplemented with 150 mM NaCl and 10% glycerol, pH 8.0.

### 2.6. Lipoprotein Labeling with Fluorescein Isothiocyanate

LDL was purified from human plasma by ultracentrifugation by adjusting plasma density with KBr (1.019 < d < 1.063). Purified LDL were labeled with fluorescein isothiocyanate (FITC) by adding 10 μL of FITC (2 mg/mL) to 1 mL of a LDL solution (1 mg/mL apoB) in 0.1 M NaHCO_3_, pH 9.0. The mixture was mixed for 2 h by slow rocking at room temperature. Unbound dye was removed by gel filtration on a Sephadex G-25 column equilibrated with PBS EDTA-free buffer. All fractions were assayed for protein content using bovine serum albumin as standard (Pierce BCA protein assay; Pierce, Thermo Fisher Scientific, Carlsbad, CA, USA).

### 2.7. Analysis of LDLR Expression and LDL Uptake by Flow Cytometry

**Figure A1:**
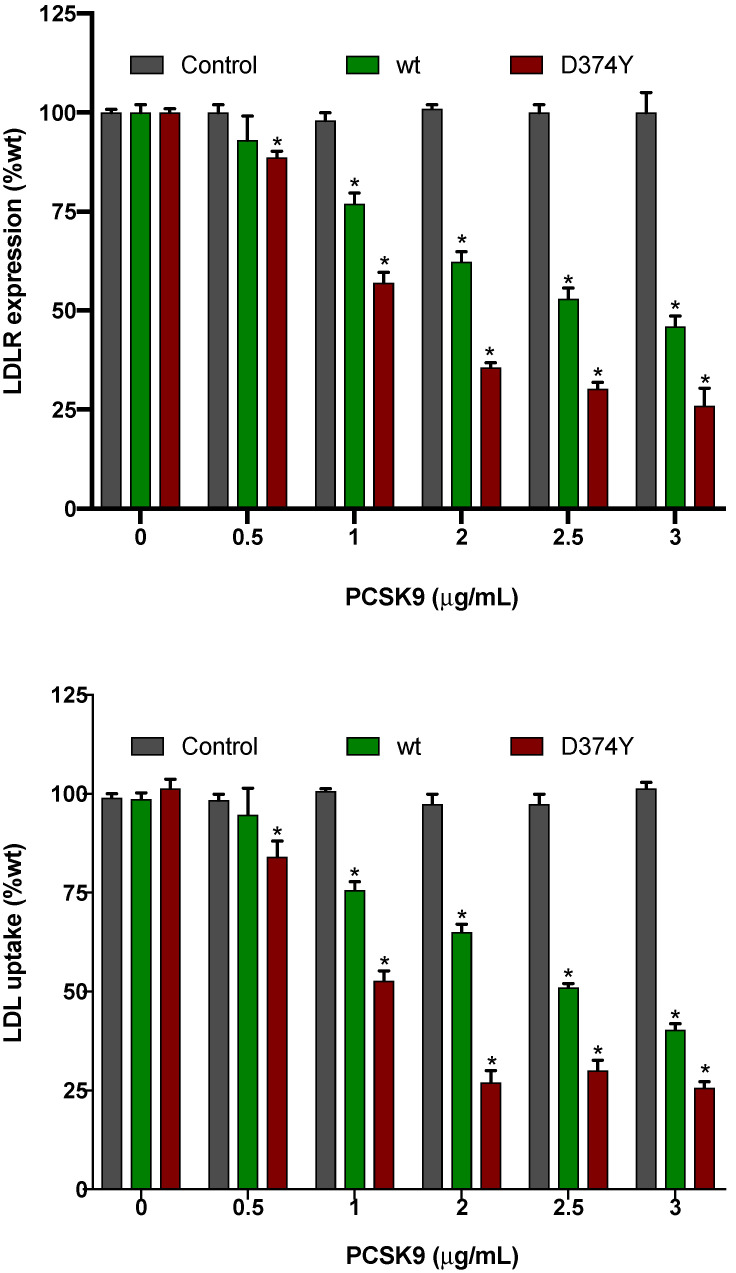
PCSK9 dose–response assay to determine optimal concentration to assess LDLR expression and LDL uptake. Purified PCSK9 was added at different concentration to the culture medium and 2 h post-addition, cells were incubated with 20 µg/mL LDL for additional 2 h; thereafter, LDLR expression and LDL uptake was determined by flow cytometry as described in the Materials and Methods. For each sample, the fluorescence of 10,000 events was acquired for data analysis at least in triplicates. Data represent the mean ± SD of three independent experiments. * *p* < 0.01 compared to 0 µg/mL.

LDLR cell surface expression was determined by flow cytometry. This was achieved on transiently transfected HEK293 cells as well as on HepG2 cells incubated for 2 h with 2 µg/mL of the purified PCSK9 variants. A dose–response assay to adjust optimal PCSK9 concentration was performed (Figure A1 in Appendix A). Cells were incubated with a mouse anti-LDLR primary antibody (clone IgG7; 1:100, 2.5 mg/L) (Cat. No.: 61087; Progen Biotechnik GmbH., Heidelberg, Germany) for 2 h at room temperature and then washed twice with PBS-1% BSA and incubated for 1 h at room temperature with Alexa Fluor 488-conjugated goat anti-mouse IgG secondary antibody (1:100) (Cat. No.: A11001; Thermo Fisher Scientific, Molecular Probes, Carlsbad, CA, USA). To determine the effect of PCSK9 variants on LDL uptake, HEK293 cells were incubated for 4 h at 37 °C with 20 µg/mL FITC-LDL. After incubation, cells were washed twice in PBS-1%BSA, fixed on 4% formaldehyde for 10 min, and washed again twice with PBS-1%BSA. To determine internalized LDL, extracellular fluorescence was quenched by adding Trypan blue solution (Sigma-Aldrich, Steinheim, Steinheim am Albuch, Germany) at a final concentration of 0.2%. Fluorescence intensities were measured by flow cytometry on a FACSCalibur™ (BD Bioscience, San Jose, CA, USA). All measurements were performed at least in triplicate, and 10,000 events were acquired for data analysis in each sample.

### 2.8. Purification of LDLR-Ectodomain

The N-terminal extracellular ectodomain of the LDLR (ED-LDLR, corresponding to 1–789 amino acids) carrying both c-myc and His tag was purified from HEK293 cells transfected with the pcDNA3.1-EC-LDLR-His plasmid, kindly provided by Prof. Leren [25]. HEK293 cells at 70–80% confluence transfected with the plasmid by the calcium phosphate method were selected in successive passages by geneticin (G-418 sulfate; Gibco, Thermo Fisher Scientific, Waltham, MA, USA) (0.5 mg/mL). For ED-LDLR expression and purification, cells were grown in Opti-MEM (Invitrogen, Thermo Fisher Scientific, Carlsbad, CA, USA) without geneticin and maintained under these conditions for 72 h. The medium was collected, supplemented with protease inhibitors (cOmplete™ EDTA-free; Roche, Merck, Darmstadt, Germany), and ED-LDLR was purified by nickel affinity chromatography and stored at −80 °C in storage buffer (50 mM Tris-HCl, 50 mM NaCl, 10% glycerol, and 0.01% Brij-35) at pH 7.5.

### 2.9. Analysis of PCSK9-LDLR EC_50_ by Solid-Phase Immunoassay

Purified ED-LDLR diluted in working buffer (10 mM Tris-HCl, pH 7.4, 50 mM NaCl, 2 mM CaCl_2_) was used to coat 96-well microtiter plates at a fixed concentration by incubation overnight at 4 °C. Plates were blocked and incubated with a serial dilution of each of the PCSK9 variants diluted in working buffer (pH 7.4) for 2 h at room temperature. Plates were washed thoroughly with working buffer containing 0.1% Tween 20 (Merck, Sigma-Aldrich, Steinheim am Albuch, Germany). Rat monoclonal anti-DYKDDDDK tag (clone L5) (Cat. No.: MA1-142; Thermo Fisher Scientific, Carlsbad, CA, USA) and peroxidase-conjugated goat anti-rat (Cat. No.: 7077S; Cell Signalling Technology^®^ Inc., Danvers, MA, USA) antibodies were used for detection [21]. 2,2′-Azino-bis (3-ethylbenzothiazoline-6-sulfonic acid) substrate solution (Merck, Sigma-Aldrich, Steinheim am Albuch, Germany) was used as a substrate, and absorbance was determined at 405 nm. All absorbance values were corrected for unspecific binding relative to maximum absorbance, and EC_50_ values were extracted from curves after fitting the data to 5-parameter logistic (5-PL) equation (SigmaPlot 13.0, Systat Software Inc., San Jose, CA, USA).

### 2.10. PCSK9 Intracellular Activity

The intracellular activity of PCSK9 was determined by analyzing the secretion of soluble LDLR in the media. This was achieved by co-transfecting PCSK9 and the ecto-domain of the LDLR, which was an approach previously validated by Strom et al. [26]. Quantifying the amount of secreted LDLR ecto-domain is an indirect measure of the intracellular anterograde trafficking of the receptor.

HEK293 stably transfected with the different variants were transiently co-transfected with ED-LDLR coding plasmid using calcium phosphate. Then, 24 h after transfection, cells were washed, and the medium was replaced by Opti-MEM for 24 h. Culture media were harvested, and cell lysates were prepared for protein quantification. Transfection efficiency was monitored as above. ED-LDLR secretion into the medium was analyzed by Western blot. Membranes were immunostained with a mouse monoclonal anti-c-myc antibody (clone 9E10) (Cat. No.: MA1-980; Invitrogen, Thermo Fisher Scientific, Carlsbad, CA, USA). A rabbit polyclonal IgG anti-GAPDH antibody (1:1000) (Cat. No: sc-26778; Santa Cruz Biotechnology Inc., Dallas, TX, USA) was used for normalization.

### 2.11. Statistical Analysis

Data are expressed as mean ± SD of at least 3 independent experiments performed in triplicate. Comparisons between groups were made using a Student’s *t*-test. Statistical significance was established for *p* values < 0.05.

## 3. Results

### 3.1. PCSK9 Activity, Expression, Maturation and Secretion

Analysis of LDLR activity can be easily assessed in transiently transfected HEK293 cells with the PCSK9 variants. Once cells are transfected, both LDLR expression and LDL uptake can be determined by flow cytometry. As shown in Figure 1B, LDLR expression increased when cells were transfected with both LOF variants (R46L, R194A), whereas it was reduced when cells were transfected with any of the GOF variants (D374Y, E32K, L108R, S127R, D129G, D129N, and R218S) compared with wild-type PCSK9. Accordingly, LDL uptake was increased when cells were transfected with LOF variants and reduced when transfected with the GOF variants compared with wild-type PCSK9 (Figure 1C).

All variants could be detected as non-processed pro-PCSK9 (immature, 75 kDa) and processed PCSK9 (mature 65 kDa) forms (Figure 1D,E) by Western blot. Both LOFs tested were undergoing the normal maturation process but were expressed less efficiently than wild-type PCSK9. Consequently, their secretion was significantly less than that of wild-type PCSK9.

Among the GOFs tested, only S127R and D129G variants showed reduced PCSK9 maturation and were less efficiently expressed than wild-type PCSK9 (Figure 1D–G). As a result, their global secretion was diminished compared to that of wild-type PCSK9.

**Figure A2:**
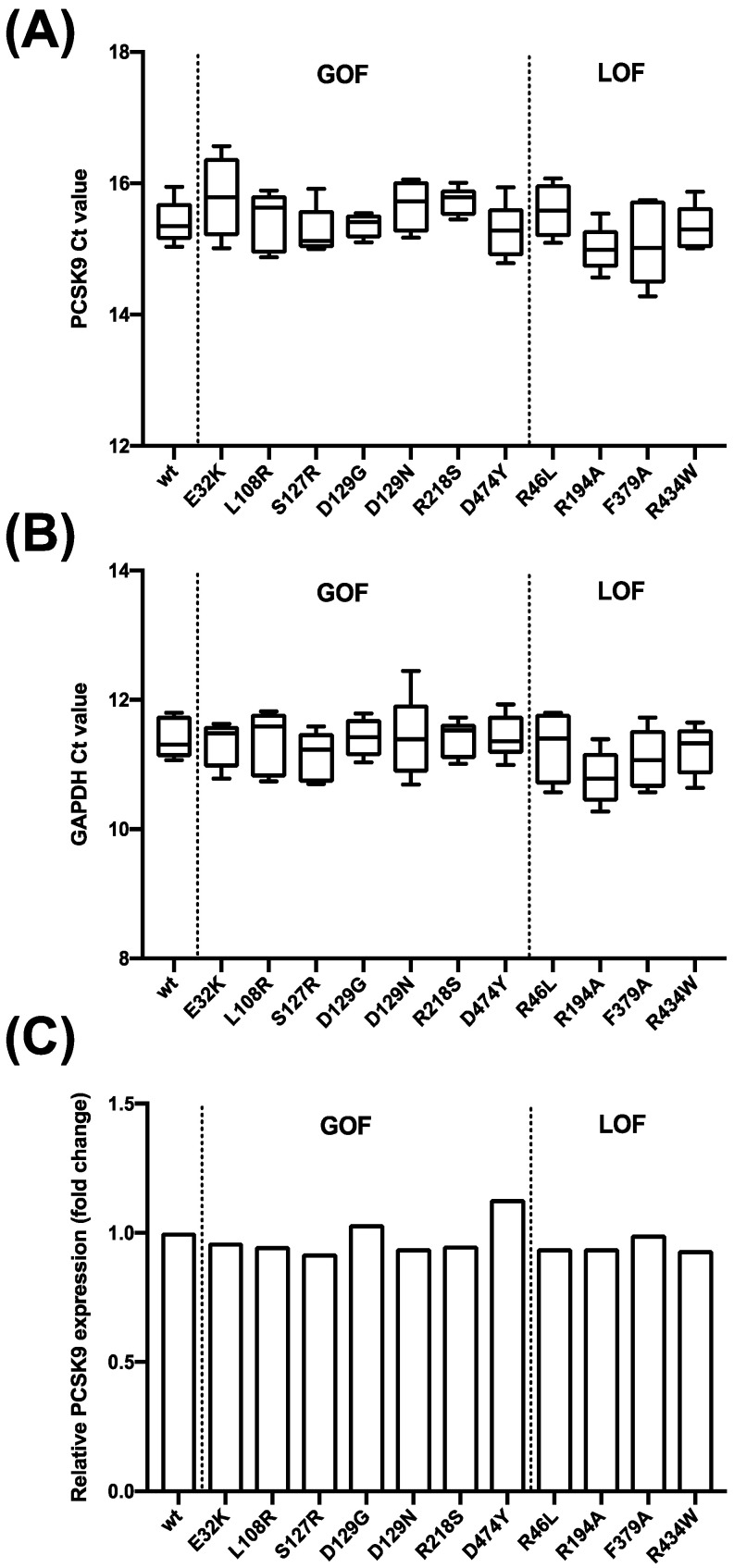
mRNA expression of PCSK9 variants in HEK293 transfected cells. (**A**) Ct values of PCSK9 mRNA; (**B**) Ct values of GAPDH mRNA, and (**C**) relative expression of PCSK9 variants to wt PCSK9. Quantitative real-time PCR (qRT-PCR) was performed in triplicate using iQ SYBR green Supermix (Bio-Rad) on a Real-Time Detection System (Bio-Rad). The mRNA levels were normalized to GAPDH mRNA as a housekeeping gene.

The protein expression and secretion of the variants determined by ELISA confirmed the observations made by Western blot (Table A1). To rule out that the difference in variants abundance could result from differences in gene expression, we ascertained that mRNA expression levels were similar for all (Figure A2).

### 3.2. PCSK9 Processing by Furin

As furin-mediated cleavage of PCSK9 abrogates its function toward the LDLR [27], variants showing the complete or partial resistance to furin cleavage are GOF [7,17,28,29,30]. Analysis of secreted PCSK9 by Western blot allows the detection of a 53 kDa band corresponding to furin-cleaved PCSK9 [5,18] together with the 65 kDa band corresponding to the non-processed form (Figure 1D,E). Among the GOF, only the R218S variant showed complete resistance to furin cleavage [7,28]. As shown in Table 1, no significant differences on 65 kDa/53 kDa ratios were detected for any of the other variants compared to wild-type PCSK9.

### 3.3. PCSK9 Extracellular Activity in HepG2 Cells

**Figure 2:**
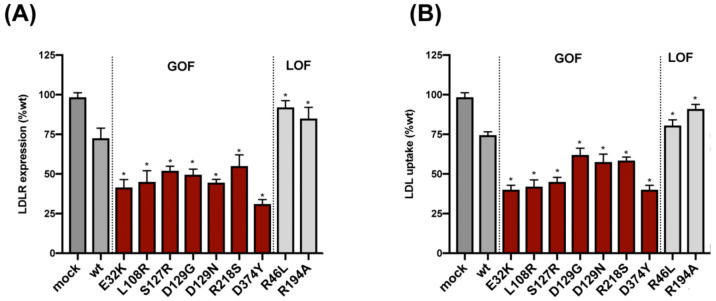
Extracellular activity of PCSK9 variants on HepG2 cells. (**A**) Cell surface LDLR expression was determined in HepG2 cells incubated with the purified variants by flow cytometry. (**B**) LDL cellular uptake was determined in HepG2 cells incubated with the purified variants by flow cytometry. Histograms represent the mean ± SD of three independent experiments performed by triplicate, * *p* < 0.01 compared to wild-type PCSK9.

Extracellular activity of PCSK9 variants was determined specifically by adding recombinant PCSK9 to HepG2 cells and then by performing the assessment of LDLR expression and LDL uptake by flow cytometry. As shown in Figure 2A,B, the addition of any one of the LOF variants onto HepG2 cells resulted in higher LDLR expression and LDL uptake, whereas the addition to any one of the GOF variants resulted in reduced LDLR expression and LDL uptake compared to wild-type PCSK9.

### 3.4. PCSK9 Affinity (EC_50_) for the LDLR

We next assessed the affinity of PCSK9 variants toward the LDLR. Binding affinities were determined by solid-phase immunoassay. R46L and R194A variants showed reduced affinity for the receptor in line with their LOF status (Table 2). Except for the R218S variant, the affinity of all GOF variants for the LDLR was significantly higher than that of wild-type PCSK9.

### 3.5. PCSK9 Intracellular Activity

**Figure 3:**
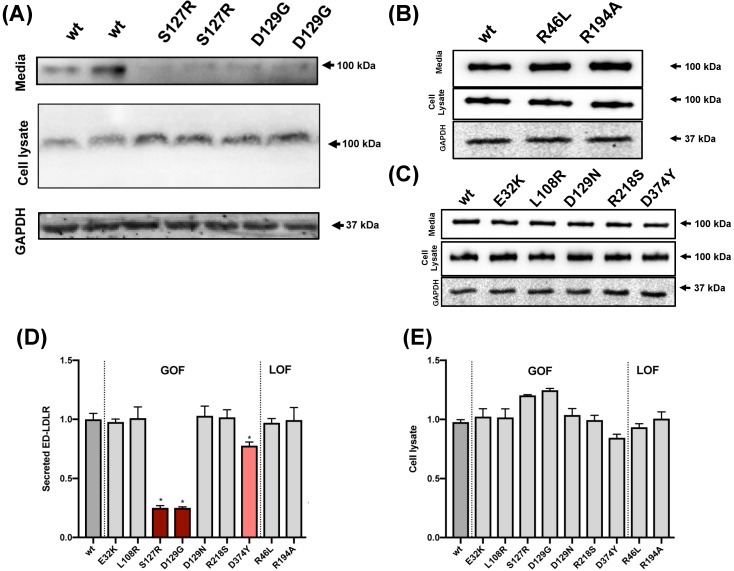
Intracellular activity of PCSK9 variants. (**A**–**C**) Representative Western blots of the intracellular activity of PCSK9 variants determined in HEK293 cells stably expressing these variants and transiently co-transfected with a plasmid allowing the expression of the ectodomain of the LDLR (ED-LDLR). The amount of the ED-LDLR was determined in cell lysates and media. (**D**) Secreted ED-LDLR was determined as the ratio between media/intracellular signals quantified by densitometry. (**E**) Quantification of intracellular ED-LDLR normalized to GAPDH. Histograms represent the mean ± SD of three independent experiments, * *p* < 0.01 compared to wild-type PCSK9.

The intracellular activity of PCSK9 was determined by analyzing the secretion of soluble LDLR in the media. This was achieved by co-transfecting PCSK9 and the ecto-domain of the LDLR. Quantifying the amount of secreted LDLR ecto-domain is an indirect measure of the intracellular anterograde trafficking of the receptor [26]. Only the medium of cells co-transfected with ED-LDLR and S127R or D129G PCSK9 variants (and to a lesser extent with the most potent D374Y variant [21]) contained reduced amounts of ED-LDLR compared with wild-type PCSK9 (Figure 3). The remaining PCSK9 variants showed similar intracellular ED-LDLR expression and secretion than wild-type PCSK9 (Figure 3).

## 4. Discussion

The aim of the present work was to set up a systematic methodology to determine the activity of PCSK9 variants and the mechanism by which these variants are GOF or LOF. PCSK9 expression, maturation, secretion, and inactivation by furin were assessed by Western blot. GOF or LOF status was determined by flow cytometry. Their precise mechanisms of action were further investigated by measuring their affinity for the LDLR and their ability to act on the receptor intracellularly (Table 3).

Our results demonstrate the usefulness of these complementary approaches that all together may seem tedious but that can easily be restricted to transient transfections followed by flow cytometry analysis for assessment of GOF and LOF status [21,22,31].

Several mechanisms contribute to the GOF or LOF status of PCSK9 variants. Although they do not appear relevant for the clinic, their assessment provides useful information to deepen our understanding of PCSK9 biology and potentially to develop new PCSK9 inhibitions therapeutic approaches. These mechanisms include (i) reduced or enhanced affinity for the LDLR, (ii) enhanced LDLR degradation intracellularly directly through the trans-Golgi network to lysosomes [11], (iii) resistance to furin-mediated cleavage which increases PCSK9 half-life [7,17,27,28,29,30], and (iv) reduced expression/secretion rate of PCSK9 variants. However, this last parameter is not the exclusive hallmark of LOF variants and therefore is not informative for GOF/LOF status determination. In addition, PCSK9 variants can concomitantly display some of these features (e.g., S127R has reduced secretion but enhanced intracellular activity and higher affinity for the receptor), and their exact classification as GOF or LOF cannot be established on the sole basis of these experiments.

Our methodology appears valid even to assess the activity of “weak” variants such as R46L, which has a relatively modest but yet significant effect on LDL receptor function [10,32,33]. Similarly, the lower affinity of R194A variant for the LDLR [34] was confirmed here by solid-phase immunoassay.

**Figure 4:**
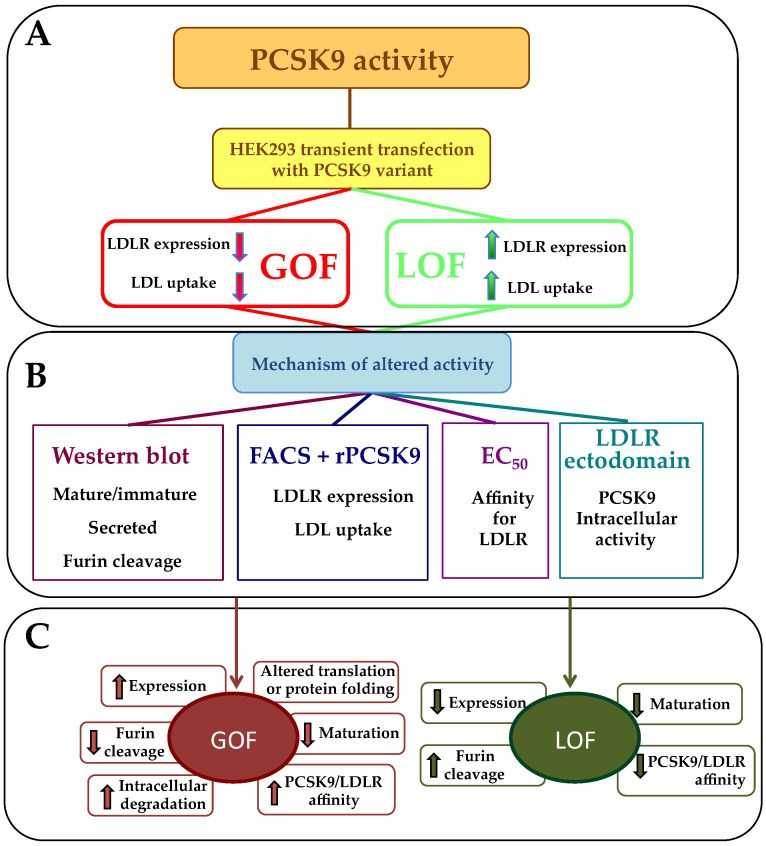
Proposed work-flow to systematically characterize a GOF or LOF PCSK9 variant activity. (**A**) Minimum methodology to assess GOF or LOF activity, (**B**) Methodology to study the mechanisms of PCSK9 variant altered activity and (**C**) mechanisms underlying GOF of LOF activities.

All the selected GOF variants showed a negative regulation of LDLR expression through different mechanisms of action. Furin cleavage resistance of R218S variant [35] and reduced maturation of S127R and D129G [36] could be assessed by Western blot. The higher binding affinities to the LDLR previously described for E32K, L108R, S127R, D129N and D374Y [30,35,36,37,38,39] was also demonstrated by solid-phase immunoassay. Figure 4 illustrates the proposed workflow to determine GOF or LOF status and the methodology required to determine the mechanism of action of each variant.

Thus, the gold standard approach to determine the status of a PCSK9 variant, beyond the genetic associations between the occurrence of a mutation and the levels of cholesterol in carriers versus non-carriers, is to undertake transient transfections of HEK293 cells with a plasmid encoding the variant under study followed by flow cytometry analysis of LDLR cell surface expression a minima. This method allows differentiating subtle changes between the activities of PCSK9 variants since the expression levels of the PCSK9 wild-type and variant transgenes are similarly very high and since HEK293 cells do not express endogenous PCSK9, thus limiting experimental noise. It is difficult to precisely define the exact contribution of these genetic alterations to the phenotype but based on the results obtained in HEK293 cells, we propose that any increase in PCSK9 activity superior to 15% may constitute a threshold to define GOF status and that any reduction superior to 25% in PCSK9 activity may constitute a threshold to define LOF status. These arbitrary cut-off values correspond to the less potent variants assessed in the present study (GOF: L108R/R218S; LOF: R46L). These investigations can easily be complemented by Western blot analyses of cell extracts and supernatants to determine the expression, maturation, and secretion levels of the PCSK9 variant under study. The intracellular activity of PCSK9 can be rather simply determined by analyzing the secretion of soluble LDLR in the media. There are inherent limitations to this approach. Thus, more refined and complex experimental protocols relying in particular on confocal microscopy and the use of gene silencing techniques have been described by Nassouri et al. [40]. For instance, such experiments could be undertaken to accurately tease out the precise mode of action of any novel mutant showing the altered secretion of soluble LDLR. Fluorescent LDL uptake experiments or assessment of LDLR expression in HepG2 incubated with purified recombinant PCSK9 variants do not appear to add sufficient additional insights and therefore should not be undertaken for diagnostic purposes. However, one limitation of the present approach is that none of these experiments take into account the possibility that some variants might variably associate with circulating lipoproteins, which has been shown to potentially reduce PCSK9 function [41]. Although very unlikely, it cannot be totally ruled out that one variant with altered/enhanced lipoprotein binding properties might fail to be properly classified as GOF/LOF using the proposed experimental approach. Another limitation of the present study relies on the limited amount of variants tested that may not represent the full spectrum of genetic defects on PCSK9 altering LDLR expression.

## 5. Conclusions

Recent guidelines recommend in vitro functional characterization as the most effective and reliable method to evaluate the pathogenicity of PCSK9 variants [42]. Our study clearly indicates that for diagnostic purposes, it is sufficient to perform transient transfections of HEK293 followed by flow cytometry analyses of LDLR cell surface expression. Nevertheless, a complete systematic and comprehensive approach is paramount to fully understand the biology of PCSK9 pathogenic variants and may in some instances be required to assign the most effective treatment targeting PCSK9 (either a monoclonal antibody that only acts on plasma PCSK9 or an antisense oligonucleotide that targets PCSK9 intracellularly) to our patients.

## Figures and Tables

**Table 1:** Ratio between mature PCSK9 (65 kDa) and furin-cleaved PCSK9 (53 kDa) bands determined by densitometry quantification of Western blot (*n* = 3).

	GOF		LOF
65 kDa/53 kDa	Mean ± s.d.	65 kDa/53 kDa	mean
wt	1.00 ± 0.2	wt	1.00 ± 0.2
E32K	0.98 ± 0.3	R46L	1.20 ± 0.1
L108R	1.01 ± 0.3	R194A	1.08 ± 0.3
S127R	1.12 ± 0.2		
D129G	0.91 ± 0.1		
D129N	0.96 ± 0.3		
R218S	0.0 0± 0.0		
D374Y	1.09 ± 0.3		

**Table 2:** EC_50_ values representing the binding affinity of PCSK9 variants to the LDLR determined by solid-phase immunoassay at pH 7.4.

	Mean ± s.d.
wt	120.6 ± 6.6
E32K	50.7 ± 4.8 **
R46L	182.0 ± 32.0 *
L108R	57.7 ± 5.6 **
S127R	50.3 ± 4.7 **
D129G	92.2 ± 5.3 *
D129N	84.0 ± 8.5 *
R194A	204.0 ± 2.3 *
R218S	112.0 ± 2.3 n.s.
D374Y	14.4 ± 0.7 **

* *p* < 0.025 compared to wt PCSK9; ** *p* < 0.01 compared to wt PCSK9; n.s. not significant compared to wt.

**Table 3:** Mechanisms leading to GOF or LOF activities of the PCSK9 variants analyzed in this study.

	LDLR Expression	LDL Uptake	Mature/Inmature PCSK9	Secreted PCSK9	Furin Cleavage	Extracellular Activity	Affinity for LDLR	Intracellular Activity	Classification
wt	-	-	-	-	-	-	-	-	**wt**
E32K	↓	↓	-	-	-	↑	↑	-	**GOF**
R46L	↑	↑	-	↓	-	↓	↓	-	**LOF**
L108R	↓	↓	-	-	-	↑	↑	-	**GOF**
S127R	↓	↓	↓	↓	-	↑	↑	↑	**GOF**
D129G	↓	↓	↓	↓	-	↑	↑	↑	**GOF**
D129N	↓	↓	-	-	-	↑	↑	-	**GOF**
R194A	↑	↑	-	-	-	↓	↓	-	**LOF**
R218S	↓	↓	-	-	↓	↑	-	-	**GOF**
D374Y	↓	↓	-	-	-	↑	↑	↑	**GOF**

Blue bar: normal activity; arrow pointing up: increased activity compared to wt; arrow pointing down: decreased activity compared to wt.

## Data Availability

The data presented in this study are available on request from the corresponding author.

## Appendix A

**Table A1:** Intracellular PCSK9 expression and secretion of the PCSK9 variants determined by ELISA in HEK293-transfected cells.

	Intracellular (ng/mg Total Protein)	Secreted (ng/mg Total Protein)
	Mean ± s.d.	Mean ± s.d.
wt	0.40 ± 0.05	61.87 ± 2.4
E32K	0.45 ± 0.03 n.s.	68.05 ± 3.5 *
R46L	0.15 ± 0.07 **	10.61 ± 5.6 **
L108R	0.40 ± 0.06 n.s.	62.01 ± 1.3 n.s.
S127R	0.23 ± 0.07 **	37.99 ± 5.5 **
D129G	0.29 ±0.02 *	38.71 ± 3.4 **
D129N	0.39 ± 0.05 n.s.	60.44 ± 3.6 n.s.
R194A	0.39 ± 0.03 n.s.	54.17 ± 5.1 n.s.
R218S	0.42 ± 0.03 n.s.	59.24 ± 4.1 n.s.
D374Y	0.31 ±0.02 **	57.72 ± 5.3 n.s.

* *p* < 0.025 compared to wt PCSK9; ** *p* < 0.01 compared to wt PCSK9, n.s. not significant compared to wt.
